# Ultradian glucocorticoid exposure directs gene-dependent and tissue-specific mRNA expression patterns *in vivo*

**DOI:** 10.1016/j.mce.2016.10.019

**Published:** 2017-01-05

**Authors:** Charlotte L. George, Matthew T. Birnie, Benjamin P. Flynn, Yvonne M. Kershaw, Stafford L. Lightman, Becky L. Conway-Campbell

**Affiliations:** a Henry Wellcome Laboratories for Integrative Neuroscience and Endocrinology, School of Clinical Sciences, University of Bristol, Dorothy Hodgkin Building, Whitson Street, Bristol, BS1 3NY, UK; b CGAT, MRC Weatherall Institute of Molecular Medicine Centre for Computational Biology, University of Oxford, John Radcliffe Hospital, Oxford, OX3 9DS, UK

**Keywords:** Ultradian rhythm, Glucocorticoids, GR, Pituitary, Prefrontal cortex, Transcription, PFC, Prefrontal cortex, PIT, Pituitary, GR, Glucocorticoid receptor, *Pomc*, Pro-opiomelanocortin, *Sgk1*, Serum/glucocorticoid regulated kinase 1, Gilz, Glucocorticoid-induced leucine zipper, hnRNA, heteronuclear ribonucleic acid, TSS, Transcriptional Start Site, GRE, Glucocorticoid Response Element, SC, subcutaneous, IV, intravenous

## Abstract

In this paper we report differential decoding of the ultradian corticosterone signal by glucocorticoid target tissues. Pulsatile corticosterone replacement in adrenalectomised rats resulted in different dynamics of *Sgk1* mRNA production, with a distinct pulsatile mRNA induction profile observed in the pituitary in contrast to a non-pulsatile induction in the prefrontal cortex (PFC). We further report the first evidence for pulsatile transcriptional repression of a glucocorticoid-target gene *in vivo*, with pulsatile regulation of *Pomc* transcription in pituitary. We have explored a potential mechanism for differences in the induction dynamics of the same transcript (*Sgk1*) between the PFC and pituitary. Glucocorticoid receptor (GR) activation profiles were strikingly different in pituitary and prefrontal cortex, with a significantly greater dynamic range and shorter duration of GR activity detected in the pituitary, consistent with the more pronounced gene pulsing effect observed. In the prefrontal cortex, expression of *Gilz* mRNA was also non-pulsatile and exhibited a significantly delayed timecourse of increase and decrease when compared to *Sgk1*, additionally highlighting gene-specific regulatory dynamics during ultradian glucocorticoid treatment.

## Highlights

• *In vivo* study of transcriptional decoding of the glucocorticoid ultradian rhythm.
• Corticosterone pulses elicit gene and tissue-specific mRNA expression patterns.
• Corticosterone pulses induce pulsatile repression of *Pomc* hnRNA in rat pituitary.
• Unlike the PFC, the pituitary exhibits pulsatile Sgk1 mRNA expression levels.
• Differential GR dynamics underlie tissue-specific transcriptional responsiveness.

## 1 Introduction

The secretion of glucocorticoid hormones from the adrenal glands of mammals is regulated by the hypothalamic-pituitary-adrenal (HPA) axis and fluctuates greatly over the course of a day. Levels of endogenous glucocorticoids (corticosterone in rats and cortisol in humans) circulating in the bloodstream not only rise in response to stress, but exhibit a well established circadian pattern in healthy unstressed individuals with glucocorticoid levels rising prior to waking and decreasing prior to sleep (Lightman and Conway-Campbell, 2010, Biddie et al., 2012). Underlying these circadian fluctuations exists a highly conserved ultradian glucocorticoid secretion pattern with pulses of endogenous glucocorticoid secreted at regular intervals (approximately 60 min in rats (Windle et al., 1998a, Windle et al., 1998b) and 60–90 min in humans (Veldhuis et al., 1989)) that arise due to an intrinsic positive feed-forward and negative feedback loop in the HPA axis (Walker et al., 2010, Walker et al., 2012).

Despite the highly dynamic nature of the endogenous glucocorticoid system, only a handful of studies have investigated how these rapid ultradian glucocorticoid fluctuations affect the transcription and mRNA expression of glucocorticoid-regulated genes (Conway-Campbell et al., 2007a, Conway-Campbell et al., 2010, Conway-Campbell et al., 2011, McMaster et al., 2011, Stavreva et al., 2015, Stavreva et al., 2009, Sarabdjitsingh et al., 2010, Morsink et al., 2006a, Morsink et al., 2006b).

Glucocorticoids diffuse readily from the blood into target cells and can bind intracellular glucocorticoid receptors (GR) and mineralocorticoid receptors (MR) which act as ligand-activated transcription factors that can interact with promoter and enhancer regions to mediate the activation or repression of glucocorticoid-regulated genes. Previous studies have demonstrated that GR responds dynamically to ultradian pulses of cortisol and corticosterone. In cell lines and in tissue, corticosterone pulses at 60 min intervals result in cyclical waves of GR:DNA binding and nascent RNA production that increase as corticosterone levels rise and rapidly decrease as corticosterone levels fall (Conway-Campbell et al., 2007a, Conway-Campbell et al., 2010, Conway-Campbell et al., 2011, Stavreva et al., 2015). However, the effects of pulsatile glucocorticoid exposure on the temporal dynamics of mRNA levels have currently been limited to cell lines (Stavreva et al., 2009, McMaster et al., 2011, Morsink et al., 2006a, Morsink et al., 2006b), and the liver (Stavreva et al., 2009) and hippocampus (Conway-Campbell et al., 2010) of rats.

GR nuclear translocation and activity is known to vary in different brain regions and cell types (Kitchener et al., 2004, de Kloet et al., 1975, McEwen et al., 1986, Spiga and Lightman, 2009). Therefore we have investigated two important glucocorticoid-target tissues, the prefrontal cortex (PFC) of the brain, and the pituitary, in which ultradian responses have not been studied. The pituitary is the primary site of glucocorticoid-dependent negative feedback and the PFC is a highly sensitive glucocorticoid-target region required for working memory and executive function (Roozendaal et al., 2004, Cerqueira et al., 2005, Butts et al., 2011, Kesner and Churchwell, 2011, McEwen and Morrison, 2013). Our *in vivo* studies into the effects of ultradian glucocorticoid exposure have, to date, been limited to the ‘hypersensitive’ (Reddy et al., 2012) glucocorticoid-responsive circadian clock gene *Period 1* (Stavreva et al., 2009, Conway-Campbell et al., 2010). We have now extended our assessment to two well-known glucocorticoid responsive genes. *Serum/glucocorticoid regulated kinase 1* (*Sgk1*) and *Tsc22d3 (Glucocorticoid-induced leucine zipper* (*Gilz*)) in the PFC, and *Pro-opiomelanocortin* (*Pomc)* in the pituitary. *Sgk1* is a widely expressed kinase that is regulated by glucocorticoids via a regulatory DNA element approximately 1 kb upstream of the transcriptional start site (TSS) (Webster et al., 1993a, Maiyar et al., 1997, Sato et al., 2008). SGK1 has been proposed to play a role in a wide range of functions, regulating both genomic and non-genomic actions and has been implicated in the regulation of many neuronal processes such as neuronal excitability, excitotoxicity, oligodendrocyte morphology, hippocampal plasticity and memory function (Tsai et al., 2002, Miyata et al., 2011, Lang et al., 2006, Lang et al., 2010). *Gilz* mRNA, transcribed from the *Tsc22d3* gene, is ubiquitously induced in the rat brain following corticosterone injections (albeit with tissue-specific variations in transcript level) and contains two glucocorticoid response element (GRE) sites within a 2500bp region upstream of the TSS (Wang et al., 2004, van der Laan et al., 2008, Yachi et al., 2007). Whilst its function in the brain remains unclear, *Gilz* is known to modulate apoptosis, with anti-proliferative effects in immune cells and the thymus (Riccardi et al., 1999, Ayroldi et al., 2007).

In this study we extend the current understanding of the effects of ultradian corticosterone exposure in living tissue, by investigating the response of these glucocorticoid-regulated genes. Using our established rat model of pulsatile corticosterone administration (Stavreva et al., 2009, Conway-Campbell et al., 2010) and a candidate gene approach, the following questions were explored: 1) Can ultradian corticosterone exposure direct unique and gene-specific regulation within the same tissue? 2) What are the dynamics of a glucocorticoid-repressible gene during ultradian corticosterone exposure? 3) Will the same gene exhibit similar or different expression profile dynamics in different tissues?

## 2 Materials and methods

### 2.1 Animals

Adult male Sprague Dawley rats (250–300 g; age 10–11 weeks) were obtained from Harlan Laboratories (Bicester, UK) and maintained under standard housing conditions with a 14:10 light/dark cycle (lights on 5.15 a.m./off 7.15 p.m.). Food and water (or saline when specified) were available *ad libitum*. All procedures were conducted in accordance with the UK Home Office guidelines and the UK Animals (Scientific Procedures) Act.

### 2.2 Surgery & pulsatile corticosterone treatment

Surgical procedures and pulsatile treatment of exogenous corticosterone were carried out as previously described (Stavreva et al., 2009, Conway-Campbell et al., 2010). Rats received a bilateral adrenalectomy (ADX) and jugular cannulation under balanced general anaesthesia (veterinary isoflurane; Merial Animal Health Ltd., UK). Post-surgery, rats received subcutaneous injections (SC) of 0.2 mg/ml Rimadyl (Carprofen 5% w/v, Benzyl alcohol 1% w/v; Pfizer Ltd., UK) diluted in sterile 10 IU/ml heparinised saline and 2.5 ml SC glucose saline (Sodium chloride 0.45% w/v and Glucose 2.5% w/v solution for infusion BP; Baxter Healthcare Ltd., UK) to aid recovery.

Animals recovered for 5 days post-surgery, during which time they received corticosterone replacement in the drinking water (0.9% saline supplemented with 15 μg/ml corticosterone (Sigma-Aldrich, UK) solubilised in 0.01% v/v absolute ethanol). Corticosterone was withdrawn 24 h prior to the experiment and replaced with 0.9% saline for drinking. On day 6, rats received up to four intravenous (IV) pulses of 100 μg corticosterone via the jugular cannula in the form of a water-soluble complex of corticosterone and 2-hydroxypropyl-β-cyclodextrin (corticosterone-HBC; C-174; Sigma-Aldrich, USA) at times 0, 60, 120 and 180 min. Blood samples were obtained by sampling directly from the cannula immediately prior to each corticosterone injection and 1 min post-injection for corticosterone radioimmunoassay (RIA). Animals were euthanized via overdose of IV sodium pentobarbital at the defined timepoints indicated in the graphs in Fig. 2, Fig. 3, Fig. 4, Fig. 5. Trunk bloods (TB) were collected and stored at −20 °C for RIA analysis and PFC and pituitaries were rapidly dissected, frozen in liquid nitrogen and stored at −80 °C.

### 2.3 Corticosterone radioimmunoassay (RIA)

Plasma samples were diluted 1 in 50 with citrate buffer (pH 3.0). Total plasma corticosterone levels were measured from blood samples with an in-house RIA as previously described (Atkinson et al., 2006, Spiga et al., 2007, Waite et al., 2012) using a specific rabbit anti-rat corticosterone primary antibody (kindly supplied by G. Makara, Institute of Experimental Medicine, Budapest, Hungary). The intra- and inter-assay coefficients of variation of the corticosterone assay have previously been established as 16.7% and 13.3%, respectively (Spiga et al., 2011).

### 2.4 Nuclear extract preparation

The nuclear extraction procedure was performed as previously described (Conway-Campbell et al., 2007a, Conway-Campbell et al., 2010, Stavreva et al., 2009, Kitchener et al., 2004, Vallone et al., 1997). Briefly, frozen PFC samples (both hemispheres) or whole pituitaries were homogenised using a Dounce homogeniser in 1 ml (PFC) or 300 μl (pituitaries) of S1 buffer (10 mM HEPES (pH7.9), 10 mM KCL, 1.5 mM MgCl_2_, 0.1 mM EDTA (pH8.0), supplemented with 2 mM NaF, 0.2 mM sodium orthovanadate, 0.5 mM DTT, and Complete™ Protease Inhibitor Cocktail (Roche Diagnostics Ltd., UK)) to release nuclei. The nuclear pellets were resuspended in 1.2 vol of cold S2 buffer (10 mM HEPES (pH 7.9), 400 mM NaCl, 1.5 mM MgCl2, 0.1 Mm EDTA (pH 8), and 5% glycerol, supplemented with 2 mM NaF, 0.2 mM Na orthovanadate, 0.5 mM DTT, and Complete™ Protease Inhibitor Cocktail) and stored in aliquots at −80 °C. Protein concentrations were assessed using a bicinchoninic acid (BCA) protein assay (Pierce, Rockford, IL).

### 2.5 Western blotting

Western blotting procedures followed the original methodology of SDS PAGE electrophoresis described by Laemmli (Laemmli, 1970) and performed as previously described (Conway-Campbell et al., 2007a, Conway-Campbell et al., 2007b, Conway-Campbell et al., 2008). For each sample 10 μg of nuclear extract was run on 7.5–15% polyacrylamide gels and transferred to polyvinylidene fluoride (PVDF) membrane (GE Healthcare UK Ltd., UK). Membranes were blocked overnight at 4 °C in 5% non-fat skimmed milk powder in Tris-buffered saline/Tween (TBST; 30 mM Tris-HCL pH7.6, 140 mM NaCl, 0.1% Tween-20) before probing with GR M-20 antibody (sc-1004, Santa Cruz) at 1:1000 (upper membrane) in 0.5% non-fat skimmed milk powder in TBST or anti-Lamin A/C (2032, Cell Signalling Technology, Boston MA) at 1:1000 (lower membrane) in TBST, both for 1 h at room temperature on a rotating platform then incubated with Horse-Radish Peroxidase (HRP) conjugated secondary antibodies (anti-rabbit IgG-HRP NA934V, GE Healthcare UK Ltd. for GR) at 1:10,000 in 5% non-fat skimmed milk powder in TBST for 1 h. Membranes were treated with enhanced chemiluminescence (ECL) reagent (ECLPlus, Biological Industries, Israel) and signal detected using enhanced chemiluminescence hyperfilm (GE Healthcare UK Ltd). Results were quantified by densitometry using an Epson perfection scanner (Epson Europe, Netherlands) in professional mode 16-bit grey-scale. Data were analysed using Image J Densitometry Software (http://imagej.nih.gov/ij) with adjusted volume OD for GR, normalised to total protein loaded and expressed as fold-change relative to the time 0 control for each tissue type.

### 2.6 RNA extraction

Total RNA from both prefrontal cortices and whole pituitaries was extracted using TRizol reagent (Invitrogen, Life technologies Ltd., UK) and using Heavy Phase-Lock-Gel tubes (5 PRIME, USA) to increase RNA recovery. The recovered supernatant was subjected to a second purification step via incubation with an equal volume of 24:1 chloroform:isoamyl alcohol (Sigma-Aldrich, UK). Samples were then processed using Qiagen RNeasy Mini Kit (Qiagen, UK) with an on-column DNAse digestion (Qiagen RNase-Free DNase kit, UK), after which RNA aliquots were stored at −80 °C.

### 2.7 RT-qPCR

RNA concentrations were quantified by a NanoDrop ND-1000 Spectrophotometer (NanoDrop Technologies, USA). All samples possessed a 260:280 ratio of 1.8–2.2 and 260:230 ratio above 1.9. cDNA conversions were performed using 1 μg of RNA with the AMV first-strand synthesis kit (Invitrogen, Life Technologies Ltd., UK) on a G-Storm GS1 thermal cycler (Gene Technologies Ltd., UK). Control samples omitting the AMV RT enzyme were run in parallel. RT-qPCR reactions were performed on the ABI Prism 7500 Sequence Detection System (Applied Biosystems, UK). *Pomc* hnRNA expression was quantified using 50 ng of cDNA input with a custom designed FAM-dye-labelled Taqman expression assay (Forward: 5′–ACTAGAGGGCAGGGATGGT-3′, Reverse: 5′–GGGAGGCCCAATGTGTGA-3′, FAM probe: 5′–CCAAGCCAGCTCCTG-3′) normalised to *β-actin* mRNA (ABI assay no. 4352931E; Applied Biosystems, UK), performed with TaqMan Universal PCR Master Mix (Applied Biosystems). *Sgk1* and *Gilz* (*Tsc22d3*) mRNA expression levels were quantified relative to *β-actin* mRNA expression using 100 ng of cDNA per replicate. Amplification reactions were performed with Power SYBR Green Master Mix (Applied Biosystems) and custom SYBR green primers at a working concentration of 2 μM (Sigma-Aldrich, UK; *Sgk1* mRNA: Forward: 5′-CGTACGACCGGACAGTGGA-3′, Reverse 5′-GATATTTGGTTTCAGCTGGAGAGG-3’; *Gilz* mRNA: Forward: 5′–CCATGGATCTAGTGAAGAATCATTTG-3′, Reverse: 3′–CCACCTCCTCTCTCACAGCATAC-5′, *β-actin* mRNA: Forward: 5′-CTTCTTGCAGCTCCTCCGTC-3′, Reverse: 5′-ATATCGTCATCCATGGCGAAC-3′). Standard curves confirmed that the amplification efficiency of all primers fell within the recommended ABI guidelines of 100 ± 10% efficiency and thus transcripts were quantified using the comparative ΔΔCt method. Dissociation curves were performed for all SYBR Green assays and used for quality control. Ct values were defined using automatic thresholds and baseline settings using Sequence Detection Software (version 1.2.3; Life Technologies Ltd., UK).

### 2.8 Statistics

Microsoft Excel 2008 for Mac (version 12.3.6) was used for raw data analysis. Graphs and statistics were performed using GraphPad Prism Version 6 for Mac OS X. All graphs and data in text are expressed as mean (*M*) ± s.e.m, with a minimum *n* number per group of 3. One-way ANOVAs were used to analyse the timecourse data, and Tukey's or Dunnett's multiple comparison post-hoc tests were then used where appropriate. Gene-specific and tissue-specific effects in the data were assessed using two-way ANOVA, followed by Tukey's or Sidak's multiple comparison post-hoc tests where appropriate.

## 3 Results

### 3.1 Pulsatile corticosterone treatment model

**Fig. 1:**
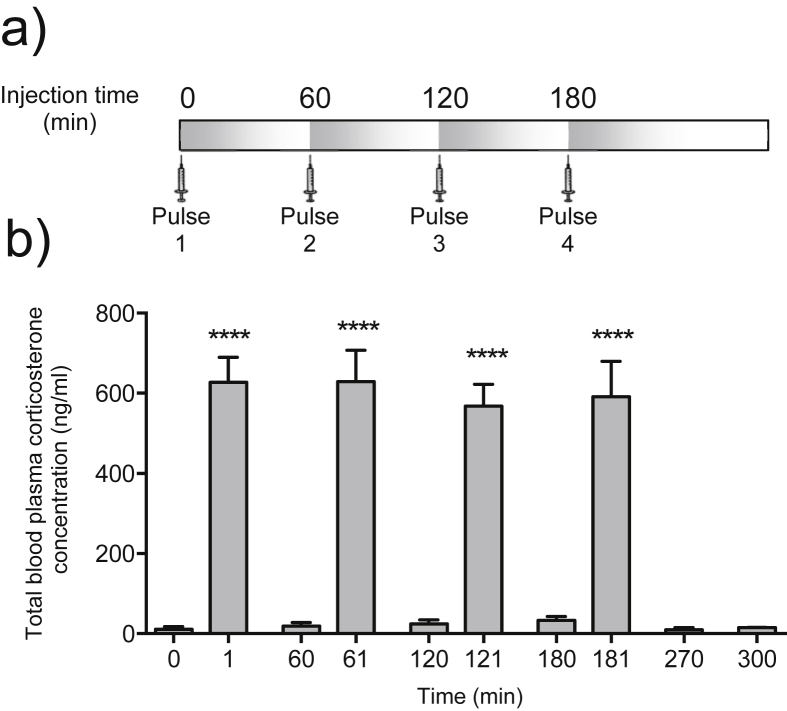
**The corticosterone profile of rats during ultradian treatment.** Administration of four IV bolus injections (‘hourly pulses’) of 100 μg exogenous corticosterone at exactly 0, 60, 120 and 180 min **(a)** results in the distinctive circulating corticosterone profile in adrenalectomised rats **(b)**. Pulsatile glucocorticoid levels in the bloodstream (measured in plasma samples by RIA) rise rapidly within 1 min of each corticosterone administration and return to baseline levels within 60 min, temporally modelling the endogenous rhythm of ultradian glucocorticoid dynamics. All data expressed as mean ± s.e.m. One-way ANOVA revealed a significant effect of time (*P* < 0.0001). Individual significant differences, determined by Dunnett's *post hoc* test with 0 min used as control, are indicated (****P < 0.0001).

Rapid rises in plasma corticosterone levels were observed 1 min after the hourly administration of each corticosterone ‘pulse’ (Fig. 1), providing a circulating corticosterone profile consistent with our previously described model of ultradian glucocorticoid replacement (Stavreva et al., 2009, Conway-Campbell et al., 2010). Plasma corticosterone cleared to basal levels within 60 min of each pulse, consistent with the 8–9 min half life of corticosterone clearance *in vivo*. This profile temporally models the hourly intervals observed with endogenous ultradian glucocorticoid secretion (Windle et al., 1998a, Windle et al., 1998b, Stavreva et al., 2009, Conway-Campbell et al., 2010), and will be the model used throughout the present study for downstream assessment of transcriptional rhythmic responsiveness in the PFC and pituitary.

### 3.2 Ultradian glucocorticoid exposure directs gene-specific mRNA expression in the PFC

**Fig. 2:**
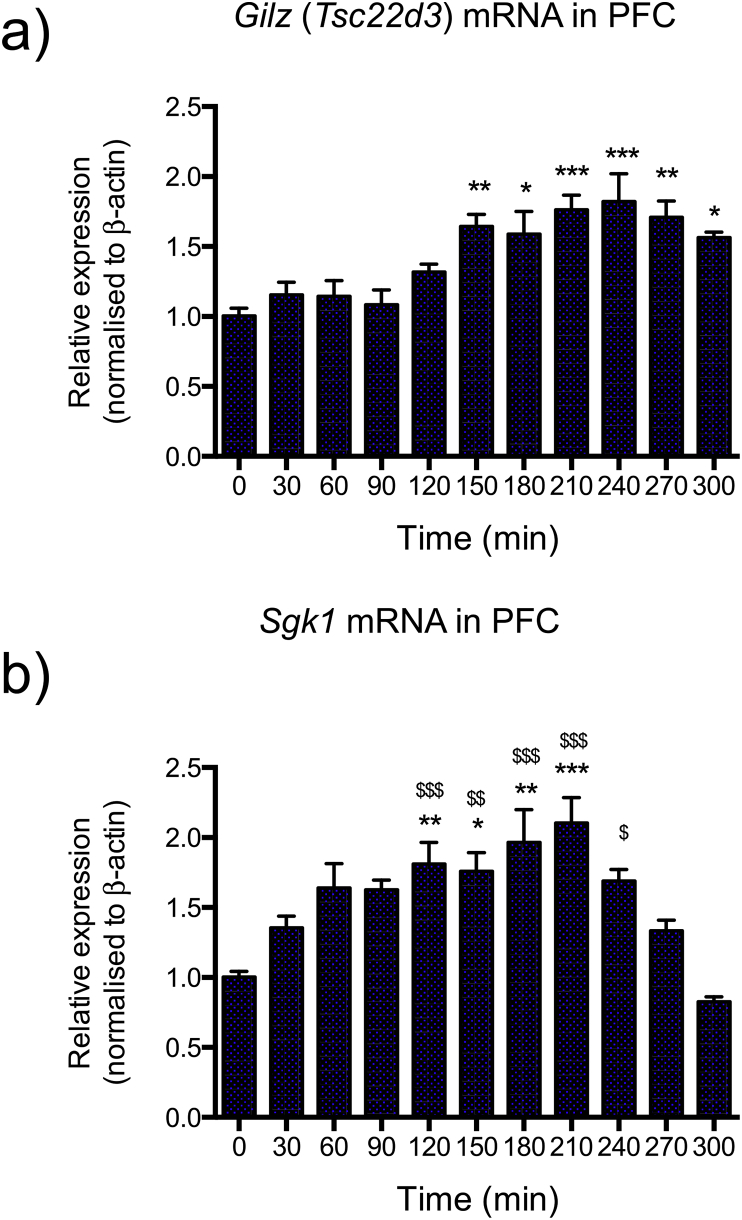
Ultradian corticosterone exposure results in differential gene-specific mRNA expression profiles for *Gilz* and *Sgk1* in the PFC. (a) Four consecutive hourly pulses of 100 μg of exogenous corticosterone resulted in a significant induction of *Gilz* mRNA in the PFC (*P* < 0.0001; One-way ANOVA) with a notably delayed induction observed. Consistent with the visibly delayed induction profile, Tukey's multiple comparison tests determined that *Gilz* mRNA levels did not reach statistical significance (relative to baseline, time 0) until 150 min. *Gilz* mRNA levels then remained significantly elevated throughout the time course including at 300 min. Asterisks above individual bars indicate significant differences compared to time 0 min (* = *P* < 0.05, ** = *P* < 0.01, *** = *P* < 0.001). In comparison, *Sgk1* mRNA (b) increased more rapidly, with a trend of increase as early as 30 min, reaching significantly elevated levels by 120 min then decreasing by 270 min. One-way ANOVA indicated the effect was significant (*P* < 0.0001), and the results of Tukey's multiple comparison tests are shown on the graph. Asterisks above individual bars indicate significant differences for each timepoint compared to baseline time 0 min (* = *P* < 0.05, ** = *P* < 0.01, *** = *P* < 0.001), and ^$^ symbols above individual bars indicate significant difference compared to time 300min (^$^ = *P* < 0.05, ^$$^ = *P* < 0.01, ^$$$^ = *P* < 0.001). All data were normalised to *β-actin* and expressed as mean ± s.e.m.

Striking differences in mRNA expression profiles for *Gilz* and *Sgk1* were apparent over the 5 h timecourse of ultradian corticosterone treatment. *Gilz* mRNA (Fig. 2a) levels remained at a low plateau during the first three corticosterone pulses, only increasing significantly above basal levels at 150 min (*M* = 1.65 ± 0.08 fold induction, *P* < 0.01), suggesting at least three consecutive corticosterone pulses were required to induce an increase in expression of this transcript. *Gilz* mRNA then remained significantly elevated throughout the timecourse up to 300 min, which is 120 min after the final pulse. In contrast, *Sgk1* mRNA (Fig. 2b) expression increased more rapidly after the first corticosterone pulse and was significantly elevated relative to baseline levels (0 min) by 120 min (*M* = 1.81 ± 0.15 fold induction, *P* < 0.01), then continued to increase until 210 min (*M* = 2.10 ± 0.18 fold induction, *P* < 0.001). At 240 min (60 min after the final corticosterone pulse) *Sgk1* mRNA levels began to rapidly fall, returning to baseline levels by 300 min (*M* = 0.83 ± 0.03 fold induction, P > 0.05).

### 3.3 Pulsatile repression of Pomc hnRNA in the pituitary

**Fig. 3:**
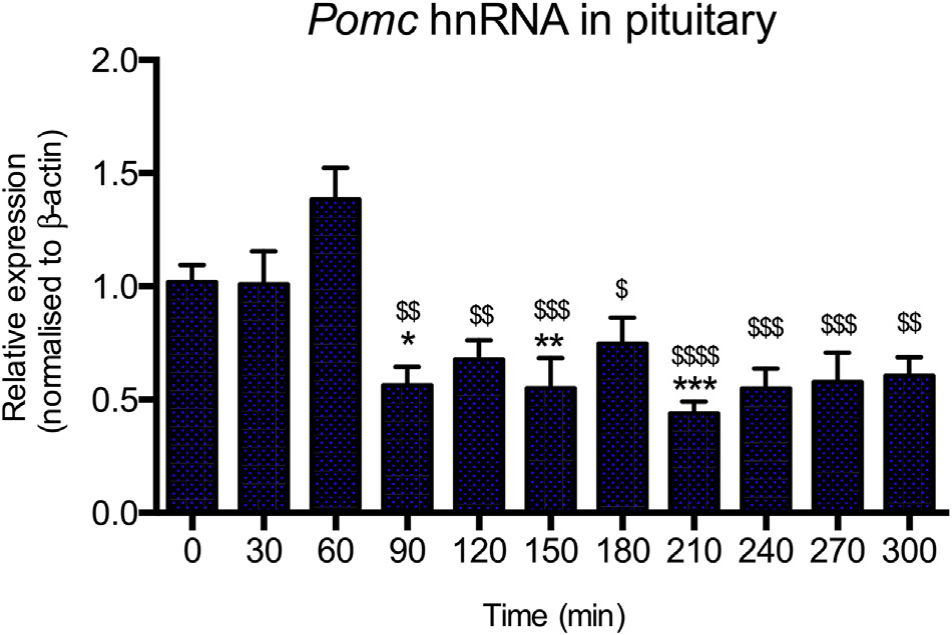
**Ultradian glucocorticoid exposure results in downregulation of *Pomc* in the pituitary**. Four consecutive hourly pulses of 100 μg of exogenous corticosterone resulted in a significant (*P* < 0.0001; One-way ANOVA) reduction of *Pomc* hnRNA in the pituitary with a notably delayed effect observed. A trend to increased *Pomc* hnRNA levels was observed at 60 min, although this was not significant (Tukey's multiple comparison post-test; *P* > 0.05). A ‘pulsatile’ repression of *Pomc* hnRNA was evident from the second pulse application. Results from Tukey's multiple comparison tests are indicated on the graph, where statistical significance was found. Asterisks on the individual timepoints of 90, 150 and 210 min indicate individual comparisons to control time 0 (* = *P* < 0.05, ** = *P* < 0.01, *** = *P* < 0.001), consistent with a pulsatile repression of *Pomc* 30 min after the consecutive corticosterone pulses administered at 60, 120 and 180 min. There was a lack of significant difference from control time 0 for times 120, 180 and 240 min, consistent with a transient repression after each pulse. Significant reduction in *Pomc* hnRNA levels at 90 min and then throughout the timecourse were evident only in the Tukey's comparisons to the 60 min timepoint, with individual results indicated by the ^$^ symbol (^$^ = *P* < 0.05, ^$$^ = *P* < 0.01, ^$$$^ = *P* < 0.001, ^$$$$^ = *P* < 0.0001). All data were normalised to *β-actin* and expressed as mean ± s.e.m.

Previous studies into ultradian glucocorticoid exposure *in vivo* have primarily focused on upregulated genes. Glucocorticoids however, can also act as negative regulators of gene expression, inhibiting the transcription of numerous important target genes to exert many of their physiological effects (De Bosscher et al., 2003). The negative feedback arm of the HPA axis for instance, involves glucocorticoids acting on pituitary corticotrophs to directly inhibit transcription of the *Pomc* gene which encodes precursors to the hormone adrenocorticotrophic hormone (ACTH) that stimulates corticosterone secretion (Birnberg et al., 1983). *Pomc* mRNA has, however, a particularly long half life (up to 24 h (Birnberg et al., 1983)) which may mask the direct transcriptional changes induced by glucocorticoids over this short time period. The nascent transcript (hnRNA) of the *Pomc* gene in the rat pituitary was therefore investigated to assess the direct transcriptional effects of pulsatile glucocorticoid exposure (Fig. 3). In contrast to the rapid *Sgk1* mRNA induction seen in the PFC (Fig. 2b) and *Per1* hnRNA seen in the liver and hippocampus (Stavreva et al., 2009, Conway-Campbell et al., 2010), no significant changes in *Pomc* hnRNA expression were detected in response to the first corticosterone pulse. In addition to a lack of downregulation of *Pomc* hnRNA in response to the first pulse, there was a trend (not significant) of an increase at 60 min. Each subsequent pulse then appeared to transiently reduce *Pomc* hnRNA, with notable decreases evident at 90 min (suppression; *M* = 0.56 ± 0.19), 150 min (suppression; *M* = 0.55 ± 0.13) and 210 min (suppression; *M* = 0.44 ± 0.05). Minor recovery of suppression was evident at 120 min (suppression; *M* = 0.68 ± 0.22), 180 min (suppression; *M* = 0.75 ± 0.25), and 240 min (suppression; *M* = 0.55 ± 0.19). One-way ANOVA confirmed that the variation over time was statistically significant (*P* < 0.0001), therefore Tukey's multiple comparison tests were then used to assess the significance of the observed ‘pulsatile’ repression of *Pomc* hnRNA. Significance at the individual timepoints 90, 150 and 210 min (Fig. 3) indicated comparisons to control time 0, and therefore supported a pulsatile repression at 30 min after consecutive corticosterone pulses. There was no significant difference for times 120, 180 and 240 min compared to time 0, presumably due to the minor recovery from repression at these times, which are exactly 60 min after each consecutive pulse. A significant reduction in *Pomc* hnRNA levels at 90 min and then throughout the timecourse (Fig. 3) were evident only in the Tukey's post-test comparisons to the 60 min timepoint. Taken together these data are consistent with a pulsatile regulation of the *Pomc* transcript at a new lower set-point, established by at least two consecutive pulses.

### 3.4 Pulsatile induction of SGK1 mRNA in the pituitary

**Fig. 4:**
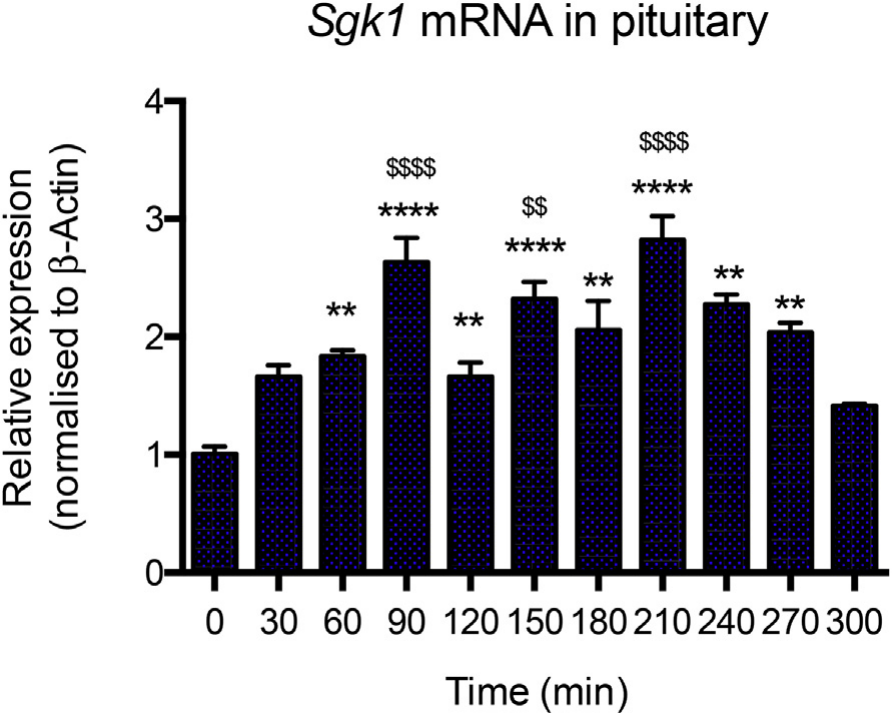
**Pulsatile *Sgk1* mRNA in the pituitary during pulsatile corticosterone exposure**. Four consecutive hourly pulses of 100 μg of exogenous corticosterone resulted in a significant (*P* < 0.0001; One-way ANOVA) induction of *Sgk1* mRNA in the pituitary. A significant upregulation of *Sgk1* mRNA occurred at 60 min in response to the first pulse. Consecutive pulses appear to then establish further transient increases in Sgk1 mRNA at times 90, 150 and 210 min i.e. 30 min after each pulse. Tukey's multiple comparison tests revealed that the highest significant differences, relative to baseline time 0, were found at times 90, 150 and 210 min represented by **** (*P* < 0.0001), while lesser inductions were evident at times 60, 120, 180, 240 and 270 min (** = *P* < 0.01). The highest significant differences relative to time 300 min (i.e. 120 min after cessation of pulsing) were found at times 90, 150 and 210 min, represented by ^$^ symbols (^$$^ = *P* < 0.01, ^$$$$^ = *P* < 0.0001). All data were normalised to *β-actin* and expressed as mean ± s.e.m.

In order to explore potential tissue-specific responses to ultradian glucocorticoid exposure, we compared the mRNA expression profile of *Sgk1* in the pituitary to its expression profile in the PFC. Similar to its early induction in the PFC, pituitary *Sgk1* mRNA (Fig. 4) increased rapidly after the first corticosterone pulse, reaching *M* = 1.83 ± 0.05-fold induction by 60 min. Pituitary *Sgk1* mRNA then continued to increase further after each consecutive corticosterone pulse reaching a maximal *M* = 2.84 ± 0.45-fold induction at 210 min. Levels then decreased within 30 min of the final pulse application, decreasing to *M* = 2.04 ± 0.16-fold at 270 min, and returning to near baseline levels of *M* = 1.41 ± 0.03-fold at 300 min.

Unlike the steady increase of *Sgk1* mRNA seen in PFC throughout the pulsatile corticosterone timecourse, pituitary *Sgk1* mRNA exhibited a distinctive pulsatile pattern that corresponded to each corticosterone pulse. Interestingly, the pulsatile pattern was only seen after the initial induction at 60 min i.e. further significant increases in *Sgk1* expression were evident at 30 min after the second, third and fourth corticosterone pulses at 60, 120 and 180 min respectively. These subsequent, relatively transient, inductions rose in response to each pulse then decreased with the falling phase of the pulse. As described for the analysis of the pulsatile repression in pituitary, Tukey's multiple comparison tests statistically confirmed extremely significant induction of *Sgk1* mRNA in pituitary relative to baseline time 0, at times 90, 150 and 210 min (*P* < 0.0001). Significant inductions, albeit to a slightly lesser extent, were also detected at times 60, 120, 180, 240 and 270 min (*P* < 0.01). Significant differences, relative to levels at time 300 min (i.e. 120 min after final injection), were again found at the times corresponding to each pulse peak i.e. 90, 150 and 210 min, as shown in Fig. 4. Taken together these data are consistent with pulsatile regulation of pituitary *Sgk1* mRNA at a raised set-point established after the first pulse.

### 3.5 Tissue-specific Sgk1 mRNA expression patterns are revealed during ultradian glucocorticoid exposure

**Fig. 5:**
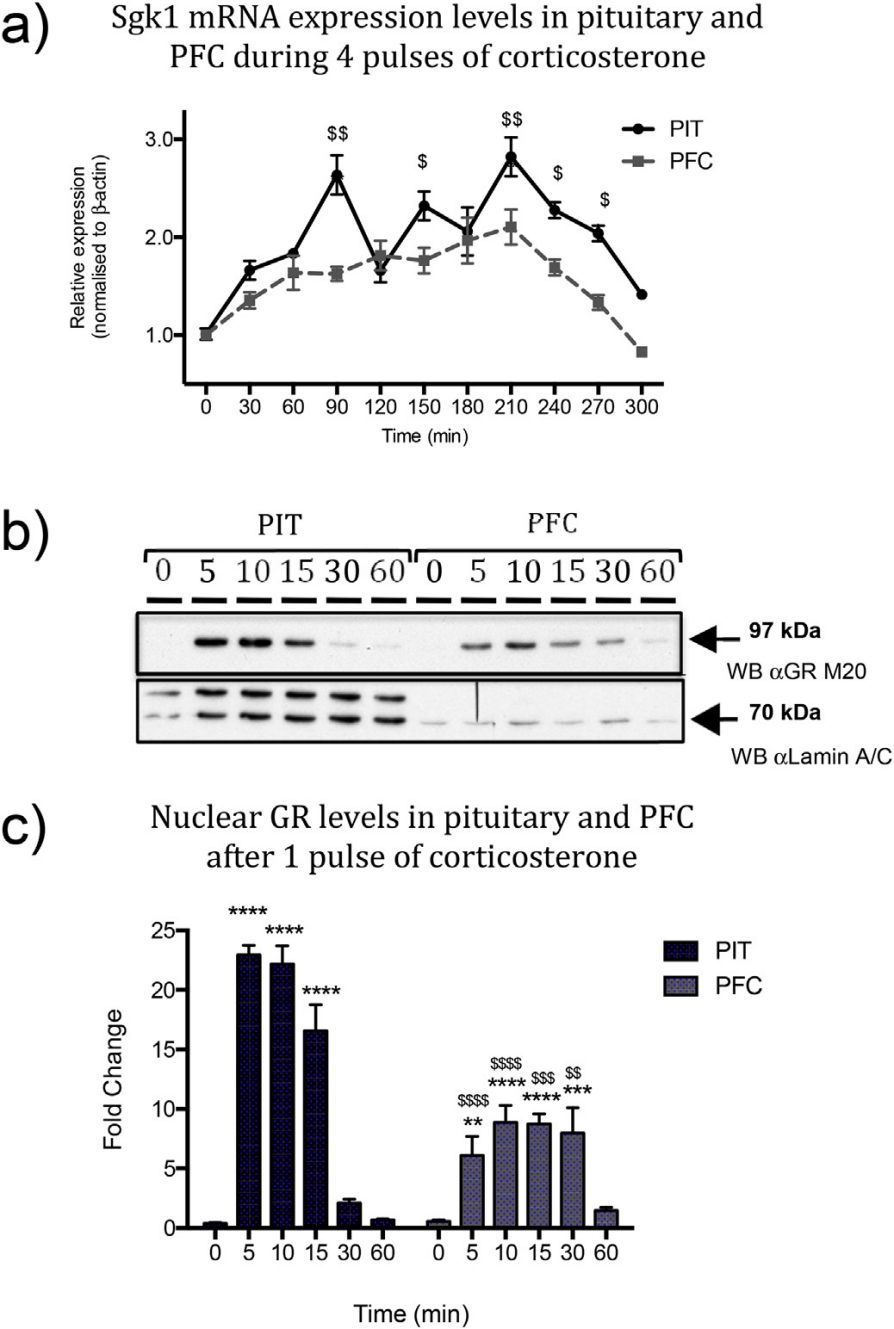
**Pituitary and prefrontal cortex exhibit differences in *Sgk1* mRNA expression profiles and GR activation dynamics during pulsatile corticosterone treatment**. Differences in *Sgk1* mRNA expression profiles between the pituitary and PFC can be visualised in the overlaid data plot **(a)**. A significant differential effect of tissue type is statistically supported by two-way ANOVA, with significant effects of both time (*P* < 0.0001) and tissue (*P* < 0.0001), with a significant interaction (*P* < 0.0001). Significant differences comparing between the pituitary and PFC at each time point are indicated in with ^$^ symbols (^$^ = *P* < 0.05, ^$$^ = *P* < 0.01; Sidak's post-tests). **(b)** Active GR is measured by the level of GR detected in the high-salt extracted nuclear fraction by western blot. Representative western blot showing a single band which resolves at approximately 97 kDa detected by the anti-GR antibody M20 (sc-1003) (upper box) in pituitary and prefrontal cortex nuclear extracts prepared from rats at times indicated after a single corticosterone pulse. Lamin A/C (nuclear extract control protein detected with antibody 2032, Cell Signalling Technology) is shown in the lower box, resolving as a double band at approximately 65 kDa and 70 kDa in the pituitary and a single band at approximately 65 kDa in the PFC. **(c)** Summary graph of western blot semi-quantitative densitometry data showing fold-change in GR detected in the nuclear fraction relative to time 0 for each tissue type. Two-way ANOVA indicated a significant effect of time after treatment (*P* < 0.0001), tissue response (*P* < 0.0001) and interaction (*P* < 0.0001). Multiple comparison test results are shown on graph. Significant differences compared to time 0 are indicated by asterisks (** = *P* < 0.01, *** = *P* < 0.001, **** = *P* < 0.0001; Tukey's post-tests), and significant differences between pituitary and PFC at each time point, are indicated by ^$^ symbols (^$^ = *P* < 0.05, ^$$^ = *P* < 0.01; Sidak's post-tests).

Fig. 5a contrasts the pulsatile *Sgk1* mRNA expression profile in the pituitary with the more continuous rise in *Sgk1* mRNA expression in the PFC. The differing tissue-specific temporal expression profiles of *Sgk1* mRNA between the PFC and the pituitary were confirmed by two-way ANOVA with highly significant effects of time (*F* (10,83) = 17.46, *P* < 0.0001), tissue (*F* (1,83) = 42.82, *P* < 0.0001), and interaction (*F* (10,83) = 3.10, *P* < 0.01). Tukey's multiple comparison test results for significant effects over time in each tissue type are shown in Fig. 3, Fig. 4, and results from comparisons between tissue types (Sidak's multiple comparison tests) are shown in Fig. 5a. Basal expression of *Sgk1* is not significantly different between the two tissues types, either at baseline (time 0) or at the time of each pulse nadir (times 60, 120 and 180 min). However, significantly higher levels of *Sgk1* mRNA are evident in the pituitary at 30 min after the second, third and forth pulse, suggesting a more dynamic range for *Sgk1* mRNA regulation in the pituitary compared to the PFC. As this might be the effect of a more dynamic range of GR response to pulsatile glucocorticoid exposure in the pituitary than the PFC, we tested for tissue-specific differences in GR activation in the two tissue types.

### 3.6 Tissue-specific decoding of ultradian glucocorticoid exposure

To assess GR activation profiles over time after a pulse of corticosterone in the two tissues, we measured GR levels in the chromatin-rich nuclear fraction prepared from the *ex vivo* tissue samples, a reliable assay of GR activity (Conway-Campbell et al., 2007a, Conway-Campbell et al., 2010, Kitchener et al., 2004, Yang et al., 1978). Western blots of the resulting nuclear extracts provided relative quantitation data for GR levels in the nuclear fraction over the timecourse after a single glucocorticoid pulse. A representative western blot (Fig. 5b) shows low nuclear GR levels at time 0 in both the pituitary and PFC. A rapid increase was evident by 5 min in both tissue types, with a much larger response in the pituitary than in the PFC. Pituitary nuclear GR levels then remained high at 10 min, decreased at 15 min and returned to baseline levels by 30 min. In contrast, nuclear GR in the PFC samples remained at elevated levels for the full 30 min before returning to baseline by 60 min. Semi-quantitative densitometry data from the full dataset was expressed as mean fold-change relative to baseline for five timepoints taken over the first hour after a single corticosterone pulse, for both pituitary and PFC (Fig. 5c). Two-way ANOVA indicated a significant effect of time (*P* < 0.0001) and tissue response (*P* < 0.0001), with a significant interaction (*P* < 0.0001). Tukey's multiple comparison post-tests confirmed significant differences compared to time 0 at times 5, 10 and 15 min for pituitary, and 5, 10, 15 and 30 min for PFC. The results of Sidak's multiple comparison tests, comparing the pituitary vs PFC GR response at each matched timepoint is indicated by the ^$^ symbols in the graph in Fig. 5c. Here, significant differences are seen at times 5, 10 and 15 min due to the significantly higher GR response in pituitary than PFC. Conversely at 30 min a significant difference is seen due to the significantly higher GR response in the PFC than pituitary. Importantly, no significant differences were detected at times 0 or 60 min. Taken together, these data indicate that the PFC exhibits quite different GR activation dynamics compared to the pituitary, with a lower initial maximal amplitude which persists for longer after the corticosterone pulse.

## 4 Discussion

### 4.1 Gene-specific differences in dynamic regulation of *Gilz* and *Sgk1* mRNA in the PFC

The differences in mRNA expression profiles of the two glucocorticoid-target genes *Gilz* and *Sgk1* in the PFC during ultradian glucocorticoid treatment are a result of: 1) a delayed induction of *Gilz* mRNA compared to *Sgk1* and 2) a more rapid decline of *Sgk1* mRNA following the cessation of pulses. Several factors that influence the rate of transcription, and stability/degradation of mRNA might explain these differences. Certainly, these intrinsic properties of transcriptional regulation and mRNA stability will enable complex digital decoding of the pulsatile glucocorticoid signal in a transcript-dependent manner.

### 4.2 Pulsatile gene regulation in pituitary

Elevated *Pomc* hnRNA levels in the early timepoints are most likely due to ADX-induced CRH and vasopressin release at the hypothalamic level, with expression levels renormalizing with corticosterone replacement. Once the new set-point is established, the rhythmic repression of *Pomc* directed by each pulse of corticosterone becomes evident. The delayed repression of *Pomc* hnRNA is also consistent with genome-wide studies in A549 cells showing that the majority of transcriptional downregulation occurs slightly later than that of transcriptional induction (Reddy et al., 2009) and with reports of a combinatorial regulatory mechanism for transcriptional repression of glucocorticoid-target genes (Cho and Kim, 2009). Consistent with this, induction of pituitary *Sgk1* occurred rapidly with increased mRNA levels detected after the first pulse. Distinctive pulsatile increases in *Sgk1* mRNA continued throughout the timecourse of pulsatile glucocorticoid exposure, establishing a higher oscillating set-point of *Sgk1* expression, which only decreased to baseline levels after the last pulse. To date the ‘gene pulsing’ phenomenon has only been described for nascent transcript production i.e. hnRNA. Therefore *Sgk1* is the first transcript found to exhibit pulsatile regulation at the mature mRNA level in our studies, presumably due to its reported short half life of 20 min (Webster et al., 1993b).

### 4.3 Tissue-specific differences between pituitary and PFC

The ability of different tissues to interpret pulses of corticosterone into either pulsatile or continuous mRNA expression levels of the same gene represents a new level of plasticity in the glucocorticoid response. Broadly speaking there are two main possibilities that could explain tissue-specific *Sgk1* mRNA expression pattern differences: 1) The transcriptional responsiveness of the tissue to ultradian corticosterone exposure, namely via the magnitude or the dynamics of tissue-specific GR responsiveness; Or 2) An effect of factors downstream of GR activation, which also regulate gene-specific temporal profiles. Examples of these downstream factors may include gene-specific chromatin modifications, or factors affecting the rate of transcription, or mRNA stability and degradation.

Tissue-specific corticosterone exposure levels determined by blood-brain barrier penetration and 11-β-HSD activity may affect the overall magnitude or duration of GR binding, as may tissue-specific differences in GR expression level or factors such as differential HSP90 activity influencing translocation. Although the kinetics of rising and falling corticosterone levels have been found to be the same in the periphery and the brain by microdialysis, free cortisol concentrations have been found to be slightly higher in the periphery (Qian et al., 2012). Furthermore, despite several studies reporting similar levels of free-corticosterone in different brain regions (Kitchener et al., 2004, Droste et al., 2008, Droste et al., 2009), region-specific GR binding and translocation profiles have been observed both within the brain (e.g. the hippocampus and PFC), and when compared to the pituitary (Kitchener et al., 2004, de Kloet et al., 1975, Spiga and Lightman, 2009, Spencer et al., 1990, Spencer et al., 1991, McEwen et al., 1976). Here we report that GR activation exhibited a greater ‘dynamic range’ in pituitary, compared to PFC, over the 60 min duration after a single corticosterone pulse. The capacity for a larger GR response in pituitary compared to PFC is already well established (Spiga and Lightman, 2009), however the marked difference in temporal dynamics between the two tissues was quite intriguing. Despite the initial higher amplitude, the pituitary GR response to a pulse of corticosterone was markedly more transient than that observed in the PFC.

Additional factors that could contribute to tissue-specific differences in *Sgk1* mRNA expression include the confounding impact of cellular heterogeneity in different tissue types, which may dampen a synchronised transcriptional response in the PFC. In particular the medial and orbital divisions of the rodent PFC respond with opposing actions to glucocorticoids and stress (McEwen et al., 2016). Therefore, it will be very interesting to be able to dissect out these two regions and assess their ultradian glucocorticoid responsiveness separately when methodological advances allow this in the future.

As disruption of ultradian glucocorticoid signalling resulting in prolonged GR activation has been linked to pathological consequences and implicated in manifestations of disease (Lightman and Conway-Campbell, 2010), the results presented here suggest that not all tissues or genes will be affected by ultradian glucocorticoid disruption in the same manner. Tissues or genes that are highly sensitive to ultradian glucocorticoid exposure (e.g. those producing pulsatile RNA expression) may be particularly prone to transcriptional disturbance during the disruption of ultradian glucocorticoid exposure by stress, disease or therapeutic glucocorticoid administration.
